# Interventions to Improve Medication Adherence in People With Hypertension: Protocol for a Systematic Review and Meta‐Analysis

**DOI:** 10.1002/hsr2.71593

**Published:** 2025-11-30

**Authors:** Kassam Hassam, Mark P. Funnell, Riya Patel, Clare L. Gillies, Kamlesh Khunti, Pankaj Gupta, Patrick Highton

**Affiliations:** ^1^ Diabetes Research Centre, Leicester General Hospital Leicester UK; ^2^ NIHR Applied Research Collaboration East Midlands, Leicester General Hospital Leicester UK; ^3^ Population Health Sciences, George Davies Centre University of Leicester Leicester UK; ^4^ NIHR Leicester Biomedical Research Centre, Leicester General Hospital Leicester UK; ^5^ Department of Cardiovascular Health Sciences University of Leicester Leicester UK; ^6^ Leicester British Heart Foundation Centre of Research Excellence UK

**Keywords:** antihypertensive agents, hypertension, medication adherence, patient outcomes, pharmacy, randomized controlled trial

## Abstract

**Background and Aims:**

Medication adherence is the extent to which a patient's behavior corresponds with the prescriber's recommendations for taking medications. Non‐adherence is particularly high in patients with hypertension, leading to poor blood pressure control, increased cardiovascular risk, and increased burden on the healthcare system. A multitude of techniques and interventions to measure and improve medication adherence have been evaluated with mixed results. This protocol outlines a systematic review and meta‐analysis of randomized controlled trials evaluating interventions aimed at improving medication adherence in people with hypertension. There will be a focus on investigating intervention impact on the duration of medication adherence, persistence, analysis of subjective versus objective medication adherence measurement techniques, the impact of patient demographics on intervention effectiveness, and the use of the Behavior Change Taxonomy to categorize behavior change interventions and facilitate their evaluation.

**Methods:**

This protocol was developed using the PRISMA‐P checklist and prospectively registered in the International Prospective Register of Systematic Reviews (PROSPERO) (CRD42024614468). A comprehensive literature search was conducted using Medline (Ovid), CINAHL and Embase electronic databases for articles published from inception to November 27th, 2024. In addition, CENTRAL was searched for articles published up to the end of 2024. Fixed and/or random‐effects meta‐analysis will be used as appropriate, according to between study heterogeneity, and the risk of bias of included studies will be assessed using the Cochrane risk of bias tool.

**Results:**

The results and findings of this review will be reported in accordance with PRISMA guidelines and published in a peer‐reviewed journal.

**Conclusion:**

The findings of this review will evaluate the evidence for interventions designed to improve medication adherence in people with hypertension. The results will provide evidence on which adherence interventions, or components of complex interventions, are effective and how they impact blood pressure control. Recommendations will be made for further research, including the design of interventions and methodology for medication adherence measurement.

## 1 Introduction

**Figure 1:**
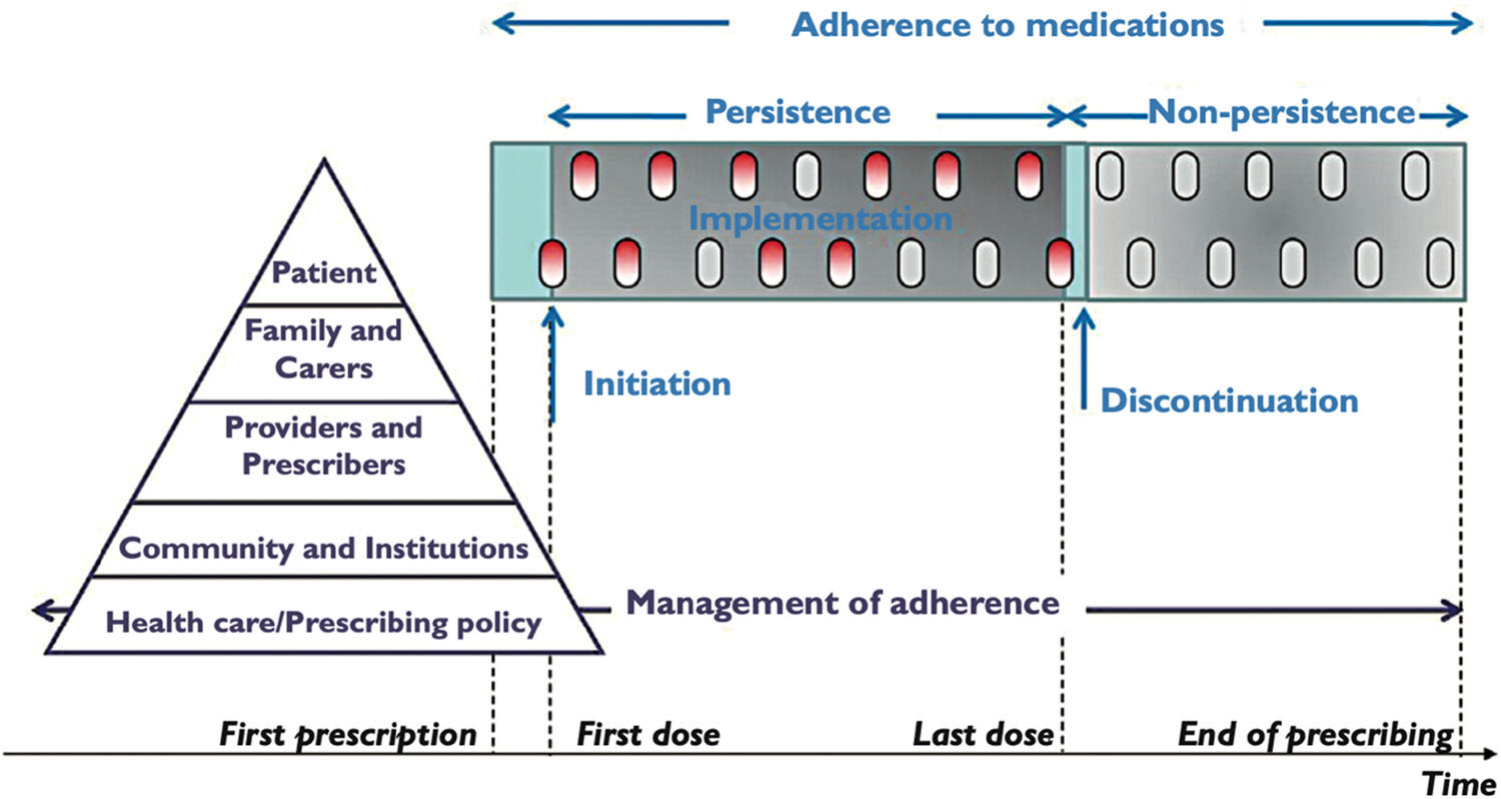
The process and taxonomy of medication adherence presented with permission [2].

Medication adherence refers to the extent to which an individual's behavior aligns with the agreed recommendations from the prescriber [1]. The process of adherence to medications is further described by phases, beginning with the first prescription and continuing until the end of prescribing, as shown in Figure 1.

Medication non‐adherence rates are typically 30%–50% in any given 6‐month period, and can be as high as 80% in those with multiple long‐term conditions (MLTCs) [3, 4]. Assuming the prescription is appropriate for the patient, non‐adherence may result in poor health outcomes and increased mortality risk for patients because the predicted treatment efficacy cannot be achieved [1, 5, 6]. Non‐adherence also increases healthcare costs if health deteriorates [7] and drives medication waste, estimated at £300 million annually in England [8].

Hypertension is the most common long‐term condition in adults over the age of 50 years living with MLTCs in the UK [9]. Globally, an estimated 1.28 billion adults aged 30–79 years have hypertension, yet fewer than half of these are diagnosed and treated [10]. As a major risk factor for cardiovascular disease, hypertension is one of the most important and treatable causes of premature death [11]. Its management typically includes antihypertensive medication and lifestyle modifications, though only half of patients receiving treatment have their blood pressure under control [10, 12].

Medication non‐adherence in patients with hypertension is particularly widespread, and the ability of patients to follow treatment plans can be compromised by a variety of patient‐related and non‐patient‐related factors. Hypertension is largely asymptomatic, therefore, patients have a tendency to associate stress symptoms with hypertension, and may discontinue medication (i.e., become non‐adherent) when stress symptoms alleviate [13].

The overall pill burden in the UK increased approximately 60% between 2002 and 2016 [14], presenting a potential barrier to adherence for hypertensive patients who typically require multiple antihypertensive drugs [15]. This pill burden is compounded as hypertension frequently coexists in combination with other long‐term conditions in adults over the age of 50 [9]. In addition, non‐adherence is implicated in up to 25%–50% of treatment‐resistant hypertension cases, complicating matters further [16, 17]. Clinicians are therefore encouraged to discuss adherence with patients at every stage before initiating additional antihypertensive drugs [11, 17].

Significant health inequalities exist in cardiometabolic disease and medication adherence. For instance, hypertension is significantly more prevalent in ethnic minority populations in the UK, and these populations have worse post‐diagnosis blood pressure control comparable to that of their white counterparts [18]. Consequently, certain ethnic minority populations, including South Asian and Black populations, are at greater cardiovascular disease (CVD) risk than White people [19, 20]. CVD prevention is therefore vital in these groups. However, antihypertensive medication non‐adherence is more common in ethnic minority populations compared to White populations [20, 21, 22, 23]. This is potentially driven by factors such as differing cultural beliefs, past healthcare experiences, communication barriers, lack of trust, perceived discrimination, and reduced health literacy [24, 25]. Irrespective of the reason, poor adherence to antihypertensive medication may lead to uncontrolled blood pressure, and consequently increased cardiovascular disease risk, poor health outcomes, and an increased burden on healthcare systems.

Previous systematic reviews and current clinical guidelines indicate a lack of strong evidence on the effectiveness of adherence interventions, which may in part be due to poor study designs and a high risk of bias (subjective adherence measurement techniques) [5, 26, 27, 28]. Complex interventions addressing multiple components of medication adherence appear to be more effective; however, unidimensional interventions, such as simplification of dosage regime, are also effective. Simplifying dosage regimens is recommended in European and American hypertension management guidelines but not explicitly in UK guidelines [27, 29, 30, 31]. Improvements in adherence to antihypertensive medication have not yet yielded a corresponding improvement in blood pressure control due to the low effect size [32]. Previous reviews indicate that studies have not collected data for each separate phase of adherence [28].

This systematic review and meta‐analysis protocol aims to identify high‐quality studies of interventions designed to improve medication adherence in patients with hypertension. This review will employ a comprehensive search strategy that intends to find and extract data pertaining to medication adherence and persistence and patient demography, such as age, sex, ethnicity, and deprivation, that have been lacking in previous reviews. It will provide an up‐to‐date examination of antihypertensive medication adherence interventions using a novel combination of techniques, including (where data allows) application of the Behavior Change Taxonomy and component network meta‐analysis, to understand the characteristics of single‐component and multicomponent interventions and their effects (overall and by separate components of the interventions) on medication adherence and blood pressure control. Additionally, it will investigate whether intervention effectiveness is influenced by patient demographics, the presence of MLTCs, and the number of medications taken. A comparison of objective versus subjective medication adherence methods will also be conducted. This protocol is published for transparency and to support methodological rigor.

## 2 Methods

This protocol has been developed using the Preferred Reporting Items for Systematic review and Meta‐Analysis Protocols (PRISMA‐P) checklist and has been prospectively registered in the International Prospective Register of Systematic Reviews (PROSPERO) (CRD42024614468). Supporting Information 1 contains a copy of the PRISMA‐P checklist that includes the page numbers upon submission of this article that correspond to each PRISMA‐P checklist item.

### 2.1 Eligibility Criteria

The PICOS (Population, Intervention, Comparison, Outcome, Study) framework was used to formulate eligibility criteria. Articles will be selected based on the inclusion and exclusion criteria in Table 1. To ensure the highest quality of data, only randomized controlled trials will be included. Additionally, careful consideration will be given to each study's objective, ensuring that only those specifically designed to impact medication adherence are selected.

**Table 1:** Inclusion and exclusion criteria.

	Inclusion	Exclusion
Population	Adult (>18 years) patients diagnosed with hypertension or prescribed medication for hypertension. Patients may also have comorbidities.	
Intervention	Improving medication adherence was an explicit aim of the intervention, either as a primary or secondary outcome.	The intervention is not designed to improve medication adherence, or medication adherence measurement is only used as an indicator for compliance to the intervention.
Comparison	A control group that received routine care and no medication adherence intervention.	Studies with no control group.
Outcome	Medication adherence measured by any means.	
Study type	Randomized controlled trial or cluster randomized trial undertaken in any setting.	Unpublished non‐peer reviewed studies. Study designs that are not randomized controlled trials.
Language	Studies written in the English language.	Studies written in languages other than English.

### 2.2 Sources and Search Strategy

PROSPERO, the Cochrane Library, and Google Scholar were searched for ongoing or recently completed systematic reviews to inform the design of this review.

A comprehensive literature search strategy was developed using a combination of relevant medical subject headings (MeSH) and keywords relating to medication adherence, hypertension, and randomized controlled trials. Search strategies from previous similar systematic reviews [26, 27], best practice guidelines [33], and additional keywords identified by the authors and an experienced academic librarian were incorporated into the search strategy. Searches looked for articles published from inception to November 27th, 2024 in Medline, CINAHL, and EMBASE databases, and to the end of 2024 in the CENTRAL database. Supporting Information 2 includes the final search strategy used for each database. Database searches will be supplemented with backward citation searching, including reference lists of relevant articles and published reviews, to identify any potential further eligible articles.

### 2.3 Data Management

Database search results will be exported from the four online databases and imported into EndNote 20 (Clarivate Analytics). Search results will be pooled and duplicates will be removed before being imported into review management software (Rayyan.ai). Articles identified by Rayyan as duplicates will be manually reviewed and deduplicated by the lead author.

Two reviewers will independently screen initial titles and abstracts for inclusion against the eligibility criteria. Full texts will be obtained for articles not excluded during title and abstract screening and will be independently screened by two reviewers. Disagreements between reviewers at any stage will be resolved through discussion or, if necessary, referral to a third reviewer. The reasons for exclusions of full‐text articles will be recorded in Rayyan. EndNote will be used to manage full‐text articles and citations for the final review results. A PRISMA flow diagram will be maintained to document the number of records at each stage of the review process. Reviewers will not be blinded to the journal titles, study authors, or institutions during screening. If an article has missing or unreported relevant data, two attempts will be made to contact the study's corresponding author(s) to obtain the data.

A bespoke data extraction form will be developed and piloted to record extracted information from the included articles. One reviewer will extract data, and a second reviewer will check the data extraction for accuracy. Sociodemographic characteristics will be extracted using the Trial Forge PRO‐EDI tool [34]. Data items to be extracted will include (where available):

- Study setting and design, including details of any theoretical frameworks applied in the design of the intervention.
- Participant data, including age, gender, ethnicity, socioeconomic, and clinical characteristics such as list of co‐morbidities and number of medications.
- Intervention details including type of intervention (e.g., educational, behavioral, pharmacological etc.), details of components, duration, and delivery method, including the role of any healthcare professionals.
- Comparison group characteristics.
- Outcomes (detailed below), including the methodology for measuring the outcome.
- Potential sources of bias, including funding sources.

### 2.4 Outcomes and Prioritization

The primary outcome for which data is to be collected is change in medication adherence, with a focus on the three distinct phases: initiation, implementation, and discontinuation [2]. Details on the methodology used to measure medication adherence will be extracted, where available, to enable a comprehensive evaluation of intervention effectiveness and enable subgroup analysis by adherence phase.

The secondary outcomes for which data are to be collected are changes in participants' blood pressure control status, and changes in the risk of cardiovascular disease and mortality. This will enable analysis of the clinical effectiveness of interventions designed to improve medication adherence.

### 2.5 Risk of Bias in Individual Studies

Risk of bias will be assessed at the outcome or results level using the Cochrane tool for risk of bias in randomized trials or the Cochrane tool for risk of bias in cluster‐randomized trials, as appropriate. Two reviewers will complete the quality assessment for each article, and disagreements between reviewers will be resolved through discussion or, if necessary, referral to a third reviewer. Studies will not be excluded due to the results of their quality assessment.

### 2.6 Data Synthesis

Tables and figures will be used to summarize the characteristics, results, and study design and methods of the included studies. Study characteristics will be analyzed and synthesized thematically, taking a narrative approach. The narrative synthesis will summarize the current evidence base, describe the interventions, and the quality of study design used.

Fixed and/or random‐effects meta‐analysis will be used to produce pooled effect sizes, as appropriate according to between study heterogeneity, which will be assessed using the Cochran's Q test, and quantified using the *I*² statistic, with thresholds of 30%, 50%, and 75% indicating low, moderate, and high heterogeneity [35]. Where necessary sensitivity analyses will be conducted to explore the impact of study quality (excluding low quality studies), and sub‐group/meta‐regression analyses will be used to explore the impact of between study heterogeneity. If complex interventions are identified, and if data are available, a component network meta‐analysis will be carried out to identify which components of the complex interventions are the most effective.

Meta‐regression and sub‐group analyses will be carried out to assess if effect sizes are associated with study‐level characteristics, including (where data is available) patient demographics (age, gender, ethnicity, socioeconomic status) and clinical characteristics (blood pressure, MLTC status, duration of hypertension, number of medications, baseline adherence status). Where the Behavior Change Techniques Taxonomy [36] has been used in the intervention design, two reviewers will code intervention descriptions to identify behavior change techniques. Outcomes will also be mapped onto the Medication Adherence Taxonomy [2, 36]. Publication bias will be assessed using Egger's test. The GRADE framework [37] will be applied to evaluate the certainty of evidence for each outcome meta‐analyzed and the strength of consequent recommendations. This will be completed independently by two reviewers, with a third reviewing helping for a consensus to be reached in the event of any disagreements.

## 3 Discussion

Hypertension is the most common long‐term condition in adults over the age of 50 years living with MLTCs in the UK [9], and medication non‐adherence in people with hypertension is particularly widespread. Medication non‐adherence not only leads to deteriorating health [6], but also contributes to significant medication waste, estimated at £300 million annually in England [8]. Addressing this issue is complex; medication adherence research is challenging due to the prevalence of bias in studies and inconclusive effect sizes found in systematic reviews. A high degree of heterogeneity between intervention studies makes it difficult to categorize interventions and draw conclusions about their effectiveness. Despite these challenges, successful interventions hold potential to improve health outcomes highlighting the need for further investigation in this area [1, 6].

This systematic review and meta‐analysis will use a novel combination of methods to provide a contemporary review of medication adherence interventions in people with hypertension. Using a comprehensive search strategy and validated tools for quality assessment and evaluation, this review aims to provide robust recommendations. It will examine the effectiveness of interventions, including the examination of components of multicomponent interventions. It will examine how subjective and objective medication adherence measurement techniques used in studies impact the effectiveness of an intervention. Additionally, the review will identify and analyze data on each phase of medication adherence, a critical stage of medication adherence in terms of patient outcomes. It will uniquely apply two established taxonomies to identify behavior change techniques and phases of adherence to determine which components or combination of components are included in effective interventions. Importantly, the review will also investigate whether the ethnic disparities observed in the clinical presentation of hypertension also extend to antihypertensive medication adherence.

We anticipate a high degree of heterogeneity between studies and will analyze this accordingly. We also expect there may be missing data on the different medication adherence phases and patient demographics which may limit certain analysis. If an overwhelming number of studies fulfill the inclusion criteria, we may need to refine and/or restrict these criteria.

The results of this review will identify the strengths and gaps in the existing literature, and the results will help to inform the design of medication adherence research and the development of adherence interventions. The findings will also provide evidence‐based, robust recommendations for clinical practice with the aim to improve patient outcomes.

## Author Contributions

**Kassam Hassam:** conceptualization, methodology, approval of final manuscript, original draft, and guarantor. **Mark P. Funnell:** conceptualization, methodology, approval of final manuscript, review and editing, and supervision. **Riya Patel:** conceptualization, methodology, approval of final manuscript, review and editing, and supervision. **Clare L. Gillies:** conceptualization, methodology, approval of final manuscript, review, and editing. **Kamlesh Khunti:** conceptualization, methodology, approval of final manuscript, review and editing, and supervision. **Pankaj Gupta:** review and editing. **Patrick Highton:** conceptualization, methodology, approval of final manuscript, review and editing, and supervision.

## Conflicts of Interest

P.G. has received funding to attend conferences or for investigator‐initiated studies from Amgen, Daiichi‐ Sankyo, Sanofi, and Servier. K.K. has acted as a consultant, speaker, or received grants for investigator‐initiated studies for Astra Zeneca, Bayer, Novo Nordisk, Sanofi‐Aventis, Servier, Lilly, and Merck Sharp & Dohme, Boehringer Ingelheim, Oramed Pharmaceuticals, Pfizer, Roche, Daiichi‐Sankyo, Applied Therapeutics, Embecta, and Nestle Health Science.

## Transparency Statement

The lead author, Kassam Hassam, affirms that this manuscript is an honest, accurate, and transparent account of the study being reported; that no important aspects of the study have been omitted; and that any discrepancies from the study as planned (and, if relevant, registered) have been explained.

## Supporting information

HTN.adherence.protocol.supporting.materials[17]_2.

## Data Availability

Please direct reasonable requests for data to the corresponding author.

## References

[1] “Adherence to Long‐Term Therapies: Evidence for Action,” World Health Organisation, published 2003.

[2] B. Vrijens , S. De Geest , D. A. Hughes , et al., “A new Taxonomy for Describing and Defining Adherence to Medications,” British Journal of Clinical Pharmacology 73, no. 5 (2012): 691–705. 22486599 10.1111/j.1365-2125.2012.04167.x PMC3403197

[3] S. Kim , K. Bennett , E. Wallace , T. Fahey , and C. Cahir , “Measuring Medication Adherence in Older Community‐Dwelling Patients With Multimorbidity,” European Journal of Clinical Pharmacology 74 (2018): 357–364. 29199370 10.1007/s00228-017-2388-y

[4] L. Foley , J. Larkin , R. Lombard‐Vance , et al., “Prevalence and Predictors of Medication Non‐Adherence Among People Living With Multimorbidity: A Systematic Review and Meta‐Analysis,” BMJ Open 11, no. 9 (2021): e044987. 10.1136/bmjopen-2020-044987 PMC8413882 34475141

[5] Excellence, N.I.f.H.a.C , “Medicines Adherence: Involving Patients in Decisions About Prescribed Medicines and Supporting Adherence (CG76),” published 2009, https://www.nice.org.uk/guidance/cg76/resources/medicines-adherence-involving-patients-in-decisions-about-prescribed-medicines-and-supporting-adherence-pdf-975631782085.

[6] C. A. Walsh , C. Cahir , S. Tecklenborg , C. Byrne , M. A. Culbertson , and K. E. Bennett , “The Association Between Medication Non‐Adherence and Adverse Health Outcomes in Ageing Populations: A Systematic Review and Meta‐Analysis,” British Journal of Clinical Pharmacology 85, no. 11 (2019): 2464–2478. 31486099 10.1111/bcp.14075 PMC6848955

[7] R. L. Cutler , F. Fernandez‐Llimos , M. Frommer , C. Benrimoj , and V. Garcia‐Cardenas , “Economic Impact of Medication Non‐Adherence by Disease Groups: A Systematic Review,” BMJ Open 8, no. 1 (2018): e016982. 10.1136/bmjopen-2017-016982 PMC5780689 29358417

[8] P. Trueman , D. G. Taylor , K. Lawson , et al., Evaluation of the Scale, Causes and Costs of Waste Medicines, (Report of DH Funded National Project, 2010).

[9] J. Valabhji , E. Barron , A. Pratt , et al., “Prevalence of Multiple Long‐Term Conditions (Multimorbidity) in England: A Whole Population Study of Over 60 Million People,” Journal of the Royal Society of Medicine 117, no. 3 (2024): 104–117. 37905525 10.1177/01410768231206033 PMC11046366

[10] “Hypertension,” World Health Organization, published 2023, https://www.who.int/news-room/fact-sheets/detail/hypertension.

[11] NICE , “Hypertension in Adults: Diagnosis and Management,” NICE Guideline [NG136], published 2023, https://www.nice.org.uk/guidance/ng136.

[12] NHS , “Health Survey for England, 2021 Part 2: Adult's Health: Hypertension,” published 2023, https://digital.nhs.uk/data-and-information/publications/statistical/health-survey-for-england/2021-part-2/adult-health-hypertension.

[13] I. J. Marshall , C. D. A. Wolfe , and C. McKevitt , “Lay Perspectives on Hypertension and Drug Adherence: Systematic Review of Qualitative Research,” BMJ 345 (2012): e3953. 22777025 10.1136/bmj.e3953 PMC3392078

[14] NHS, O.o.N.S, “Prescriptions Dispensed in the Community—Statistics for England, 2002–2012,” NHS, published 2013, https://digital.nhs.uk/data-and-information/publications/statistical/prescriptions-dispensed-in-the-community/prescriptions-dispensed-in-the-community-statistics-for-england-2002-2012.

[15] B. Vrijens , S. Antoniou , M. Burnier , A. de la Sierra , and M. Volpe , “Current Situation of Medication Adherence in Hypertension,” Frontiers in Pharmacology 8 (2017): 100. 28298894 10.3389/fphar.2017.00100 PMC5331678

[16] E. L. Schiffrin and N. D. L. Fisher , “Diagnosis and Management of Resistant Hypertension,” BMJ 385 (2024): e079108. 38897628 10.1136/bmj-2023-079108

[17] H. Durand , P. Hayes , E. C. Morrissey , et al., “Medication Adherence Among Patients With Apparent Treatment‐Resistant Hypertension: Systematic Review and Meta‐Analysis,” Journal of Hypertension 35, no. 12 (2017): 2346–2357. 28777133 10.1097/HJH.0000000000001502

[18] F. P. Cappuccio , A. Barbato , and S. M. Kerry , “Hypertension, Diabetes and Cardiovascular Risk in Ethnic Minorities in the UK,” British Journal of Diabetes & Vascular Disease 3, no. 4 (2003): 286–293.

[19] S. H. Wild , C. Fischbacher , A. Brock , C. Griffiths , and R. Bhopal , “Mortality From All Causes and Circulatory Disease by Country of Birth in England and Wales 2001–2003,” Journal of Public Health 29, no. 2 (2007): 191–198. 17456532 10.1093/pubmed/fdm010

[20] H. M. Holmes , R. Luo , J. T. Hanlon , L. S. Elting , M. Suarez‐Almazor , and J. S. Goodwin , “Ethnic Disparities in Adherence to Antihypertensive Medications of Medicare Part D Beneficiaries,” Journal of the American Geriatrics Society 60, no. 7 (2012): 1298–1303. 22702464 10.1111/j.1532-5415.2012.04037.x PMC3396714

[21] M. M. Donneyong , M. A. Fischer , M. A. Langston , et al., “Examining the Drivers of Racial/Ethnic Disparities in Non‐Adherence to Antihypertensive Medications and Mortality Due to Heart Disease and Stroke: A County‐Level Analysis,” International Journal of Environmental Research and Public Health 18, no. 23 (2021): 12702. 34886429 10.3390/ijerph182312702 PMC8657217

[22] A. Gu , Y. Yue , R. P. Desai and E. Argulian , “Racial and Ethnic Differences in Antihypertensive Medication Use and Blood Pressure Control Among US Adults With Hypertension: The National Health and Nutrition Examination Survey, 2003 to 2012,” Circulation: Cardiovascular Quality and Outcomes 10, no. 1 (2017): e003166. 28096206 10.1161/CIRCOUTCOMES.116.003166

[23] S. V. Eastwood , A. D. Hughes , L. Tomlinson , et al., “Ethnic Differences in Hypertension Management, Medication use and Blood Pressure Control in UK Primary Care, 2006–2019: A Retrospective Cohort Study,” Lancet Regional Health—Europe 25 (2023): e100557. 10.1016/j.lanepe.2022.100557 PMC9929586 36818236

[24] Y. L. Cuffee , J. L. Hargraves , M. Rosal , et al., “Reported Racial Discrimination, Trust in Physicians, and Medication Adherence Among Inner‐City African Americans With Hypertension,” American Journal of Public Health 103, no. 11 (2013): e55–e62. 24028222 10.2105/AJPH.2013.301554 PMC3828720

[25] F. Alhomoud , S. Dhillon , Z. Aslanpour , and F. Smith , “Medicine Use and Medicine‐Related Problems Experienced by Ethnic Minority Patients in the United Kingdom: A Review,” International Journal of Pharmacy Practice 21, no. 5 (2013): 277–287. 23418849 10.1111/ijpp.12007

[26] R. Nieuwlaat , N. Wilczynski , T. Navarro , et al., “Interventions for Enhancing Medication Adherence,” Cochrane Database of Systematic Reviews 2014, no. 11 (2014): eCD000011. 10.1002/14651858.CD000011.pub4 PMC7263418 25412402

[27] K. Schroeder , T. Fahey , and S. Ebrahim , “Interventions for Improving Adherence to Treatment in Patients With High Blood Pressure in Ambulatory Settings,” Cochrane Database of Systematic Reviews 2010, no. 1 (2010): eCD004804. 10.1002/14651858.CD004804 PMC9036187 15106262

[28] V. S. Conn , T. M. Ruppar , J. A. D. Chase , M. Enriquez , and P. S. Cooper , “Interventions to Improve Medication Adherence in Hypertensive Patients: Systematic Review and Meta‐Analysis,” Current Hypertension Reports 17, no. 12 (2015): 94. 26560139 10.1007/s11906-015-0606-5 PMC5662945

[29] S. T. Simon , V. Kini , A. E. Levy , and P. M. Ho , “Medication Adherence in Cardiovascular Medicine,” BMJ 374 (2021): n1493. 34380627 10.1136/bmj.n1493

[30] G. Mancia , R. Kreutz , M. Brunström , et al., “2023 ESH Guidelines for the Management of Arterial Hypertension: The Task Force for the Management of Arterial Hypertension of the European Society of Hypertension: Endorsed by the International Society of Hypertension (ISH) and the European Renal Association (ERA),” Journal of Hypertension 41, no. 12 (2023): 1874–2071. 37345492 10.1097/HJH.0000000000003480

[31] P. K. Whelton , R. M. Carey , W. S. Aronow , et al., “2017 Acc/Aha/Aapa/Abc/Acpm/Ags/Apha/Ash/Aspc/Nma/Pcna Guideline for the Prevention, Detection, Evaluation, and Management of High Blood Pressure in Adults,” Journal of the American College of Cardiology 71, no. 19 (2018): e127–e248. 29146535 10.1016/j.jacc.2017.11.006

[32] V. S. Conn , T. M. Ruppar , and J.‐A. D. Chase , “Blood Pressure Outcomes of Medication Adherence Interventions: Systematic Review and Meta‐Analysis,” Journal of Behavioral Medicine 39 (2016): 1065–1075. 26969094 10.1007/s10865-016-9730-1 PMC5018410

[33] B.M.J. Best Practice, “Study Design Search Filters,” https://dev-bestpractice.bmjgroup.com/info/toolkit/learn-ebm/study-design-search-filters/.

[34] “PRO EDI Participant Characteristics Table", 2024, https://www.trialforge.org/trial-diversity/pro-edi-improving-how-equity-diversity-and-inclusion-is-handled-in-evidence-synthesis/.

[35] J. P. T. Higgins , S. G. Thompson , J. J. Deeks , and D. G. Altman , “Measuring Inconsistency in Meta‐Analyses,” BMJ 327, no. 7414 (2003): 557–560. 12958120 10.1136/bmj.327.7414.557 PMC192859

[36] S. Michie , M. Richardson , M. Johnston , et al., “The Behavior Change Technique Taxonomy (v1) of 93 Hierarchically Clustered Techniques: Building An International Consensus for the Reporting of Behavior Change Interventions,” Annals of Behavioral Medicine 46, no. 1 (2013): 81–95. 23512568 10.1007/s12160-013-9486-6

[37] G. H. Guyatt , A. D. Oxman , G. E. Vist , et al., “GRADE: An Emerging Consensus on Rating Quality of Evidence and Strength of Recommendations,” BMJ 336, no. 7650 (2008): 924–926. 18436948 10.1136/bmj.39489.470347.AD PMC2335261

